# A Potential Nine-lncRNAs Signature Identification and Nomogram Diagnostic Model Establishment for Papillary Thyroid Cancer

**DOI:** 10.3389/pore.2022.1610012

**Published:** 2022-02-23

**Authors:** Jin-Ming Yao, Jun-Yu Zhao, Fang-Fang Lv, Xue-Bo Yang, Huan-Jun Wang

**Affiliations:** ^1^ Department of Endocrinology and Metabology, The First Affiliated Hospital of Shandong First Medical University and Shandong Provincial Qianfoshan Hospital, Jinan, China; ^2^ Shandong Key Laboratory of Rheumatic Disease and Translational Medicine, Jinan, China; ^3^ Shandong Institute of Nephrology, Jinan, China; ^4^ Department of Endocrinology and Metabology, The 960th hospital of the PLA Joint Logistics Support Force, Jinan, China; ^5^ Beijing Splinger Institute of Medicine, Jinan, China

**Keywords:** risk score, WGCNA, papillary thyroid cancer, lncRNAs, LASSO

## Abstract

The purpose of our current study was to establish a long non-coding RNA(lncRNA) signature and assess its prognostic and diagnostic power in papillary thyroid cancer (PTC). LncRNA expression profiles were obtained from the Cancer Genome Atlas (TCGA). The key module and hub lncRNAs related to PTC were determined by weighted gene co-expression network analysis (WGCNA) and LASSO Cox regression analyses, respectively. Functional enrichment analyses, including Gene Ontology and Kyoto Encyclopedia of Genes and Genomes (KEGG) and gene set enrichment analysis were implemented to analyze the possible biological processes and signaling pathways of hub lncRNAs. Associations between key lncRNA expressions and tumor-infiltrating immune cells were identified using the TIMER website, and proportions of immune cells in high/low risk score groups were compared. Kaplan-Meier Plotter was used to evaluate the prognostic significance of hub genes in PTC. A diagnostic model was conducted with logistic regression analysis, and its diagnostic performance was assessed by calibration/receiver operating characteristic curves and principal component analysis. A nine-lncRNAs signature (SLC12A5-AS1, LINC02028, KIZ-AS1, LINC02019, LINC01877, LINC01444, LINC01176, LINC01290, and LINC00581) was established in PTC, which has significant diagnostic and prognostic power. Functional enrichment analyses elucidated the regulatory mechanism of the nine-lncRNAs signature in the development of PTC. This signature and expressions of nine hub lncRNAs were correlated with the distributions of tumor infiltrating immune cells. A diagnostic nomogram was also established for PTC. By comparing with the published models with less than or equal to nine lncRNAs, our signature showed a preferable performace for prognosis prediction. In conclusion, our present research established an innovative nine-lncRNAs signature and a six-lncRNAs nomogram that might act as a potential indicator for PTC prognosis and diagnosis, which could be conducive to the PTC treatment.

## Introduction

Thyroid cancer (TC) is well-known as a common endocrine malignancy, which induces about 40,000 deaths worldwide annually [1, 2]. Papillary thyroid cancer (PTC) is the most frequent subtype of TC, and approximately 90% of TC patients fall under the category of PTC [3]. Recent therapeutic strategies for PTC patients mainly include surgical resection and radioactive iodine therapy [4, 5]. Genetic mutation and environmental exposure are major risk factors of PTC [6]. Despite more than 90% of PTC patients having satisfactory overall survival, a small proportion (about 10%) of patients with PTC have high recurrence and metastasis rates, which induces aggressive diseases and dismal prognosis [7]. Thus, it is urgently needed to screen cancer-specific factors and establish a prognostic nomogram for PTC patients’ prognosis, thereby developing a potential and accurate risk evaluation mechanism.

Non-coding RNA (ncRNA), as a type of RNA, does not code for protein but holds an enzymatic, structural, or regulatory role. Based on its transcript length, it can be classified into either small or long ncRNA. microRNA (miRNA), as the most acknowledged class of short ncRNA, is involved in the specific regulation of its target messenger RNAs (mRNAs) through inhibiting translation or inducing degradation [8]. Long non-coding RNAs (lncRNAs), as a novel class of ncRNAs, comprise more than 200 nucleotides and encode no protein [9]. In spite of having no recognized protein-coding capacity, lncRNAs are proven to have diversely structural and functional impacts in manifold processes. Data from multiple studies has implied that the influences of lncRNAs and their mechanisms of gene expression and regulation may be more extensive and complicated than those of miRNAs [10–12]. LncRNAs are expressed in many tissues *in vivo*, and their expression levels are much lower than protein coding genes, but their tissue specificity is much higher than that of protein coding genes, which makes them potentially advantageous as biomarkers with highly specific diagnostic effects [13].

Emerging evidence has suggested that lncRNAs are implicated in strings of physiological or/and pathological processes, including cell viability, apoptosis, transcriptional regulation and tumorigenesis, through regulating the gene expression at the post-transcriptional level [14, 15]. Importantly, lncRNAs are considered as oncogenic factors or tumor-suppressors in the development of PTC. For example, lncRNA TUG1 contributes to cell motility in PTC cells via targeting miR-145 [16]. Low expression of lncRNA EMX2OS can be used to predict the recurrence-free survival of PTC [17]. Wang et al. have indicated that up-regulation of lncRNA SLC26A4-AS1 could repress the epithelial-mesenchymal transition (EMT) process through mediating the MAPK signaling in PTC [18]. LncRNA DGCR5 has been identified as a tumor inhibitor in PTC [19].

The Cancer Genome Atlas (TCGA), an open source database, contains large-scale genome sequences that could be used to map genomic curves of all kinds of human cancers [20]. Development of omics technology has contributed to the diagnosis and treatment of cancers in a systematic approach [21]. Recently, a method that commonly utilized microarray data for the express profiling of lncRNAs has been well-constructed, which might advance the therapeutic measures for human cancers [22, 23]. A unique prognostic nomogram has been established based on the five-lncRNAs signature in clear cell renal cell carcinoma [24]. Moreover, a five-gene signature has been identified with a prognostic nomogram for PTC [25]. This kind of prognostic model has been considered as an important tool for risk evaluation in tumors with the consideration of roles of diverse genes on overall survival of cancer patients. However, the optimal biomarkers for PTC prognosis still have not been well characterized.

Here, we attempted to construct a tumor-specific lncRNA signature through analyzing the RNA-seq derived from TCGA database. According to the instruction of Cox regression analysis and WGCNA, a nine-lncRNAs signature with significant prognostic value in PTC was identified. Clinical association of it was confirmed through a Chi-square test. ROC curves, Kaplan-Meier (KM) plotter, and analysis with the help of TIMER website were performed to measure the importance of nine hub lncRNAs in PTC. Through logistic regression, a diagnostic model was established, whose predictive value was determined with calibration/ROC curves and PCA analysis. In short, the present investigation provides a favorable model for predicting PTC prognosis and contributes to elucidating the mechanism of patient poor prognosis.

## Materials and Methods

### Data Obtained and Analysis

Expression profiles were obtained from TCGA (https://cancergenome.nih.gov/). Patients with primary PTC were selected. Samples with incomplete clinical data and a follow-up time of <30 days were removed and finally the first group of 448 samples was obtained (Table 1 for detailed clinical information), namely the tumor group; and the second group that contained 56 normal samples, was named the normal group. The age and gender of PTC and normal control samples were matched (Supplementary Table S1).

**TABLE 1:** Summary of clinical characteristics of PTC patients.

Characteristic	N (448)
**Age**	
<45/≥45	196/252
**Gender**	
Female/Male	331/117
**Vital status**	
Alive/dead	433/15
**Pathological stage**	
I/II/III/IV	248/52/100/48
**T stage**	
T1/T2/T3/T4	134/147/147/20
**M stage**	
M0/M1	441/7
**N stage**	
N0/N1	240/208

PTC, papillary thyroid cancer; T, primary tumor; M, distant metastasis; N, lymph node metastasis.

Perl 5.0 (http://www.perl.org/) was applied for background correction and normalization of the lncRNAs expression profiles that we harvested. These downloaded lncRNAs were annotated by using Ensembl (http://www.ensembl.org/index.html), and then the differentially expressed lncRNAs (DElncRNAs) in PTC were identified using the R software “edgeR” package (http://www.bioconductor.org/packages/release/bioc/html/edgeR.html) with the criterion of |log fold change (FC)| ≥ 1 and *p* < 0.05 [26].

### WGCNA

WGCNA was employed to build a co-expression network using the 1,601 DElncRNAs that have been screened and 448 samples with complete clinical information (survival time, survival status, age, sex, pathological stage, primary tumor (T) stage, distant metastasis(M) stage, and lymph node metastasis (N) stage, etc.) [27]. After evaluating the quality of the expression matrix, we employed the WGCNA package in R to filter the soft threshold and choose *β* = 4 for the scale-free network construction. By setting min Module Size as 30 and MEDissThres as 0.25, key modules were determined.

### Establishment of lncRNAs Signature in PTC

The LASSO Cox regression model was established to target the genes evidently related with prognosis of PTC patients [28]. DElncRNAs with *p* < 0.05 were classified into two types, risky lncRNAs (hazard ratio (HR) for death > 1) and protective lncRNAs (HR for death < 1). Due to the linear combination of the expression level of lncRNAs multiplied regression coefficient derived from the univariate Cox regression analysis, the prognostic signature was established by multivariate Cox regression analysis: Risk Score = exp_lncRNA1_×beta_lncRNA1_ + exp_lncRNA12_ × beta_lncRNA2_ + exp_lncRNA3_ × beta_lncRNA3_ + exp_lncRNA4_ × beta_lncRNA4_ + exp_lncRNA5_ × beta_lncRNA5_+…+ exp_lncRNAn_ × beta_lncRNA1n_. The “exp” represents the standardized expression of each identified hub lncRNA and the “beta” was determined by multivariate Cox regression analysis.

### Assessment of the Nine-lncRNAs Signature

According to the median value of risk score, PTC patients were assigned into high risk (*n* = 224) and low risk (*n* = 224) groups. KM method was utilized to predict the prognosis of PTC patients in high- and low-risk groups. The univariate and multivariate Cox regression analyses were performed to detect whether this lncRNAs signature could act as an independent factor for prognostic prediction of PTC patients. A ROC curve was plotted to evaluate the accuracy of the nine-lncRNAs signature in PTC patients. Moreover, a prognostic nomogram of nine-lncRNAs signature was conducted to predict 1-, 2-, and 3-years overall survival rates.

### Functional Enrichment Analysis

Gene Ontology (GO) and Kyoto Encyclopedia of Genes and Genomes (KEGG) analyses were implemented to estimate the roles of the co-expressed genes of nine hub lncRNAs. Pearson correlation analyses were implemented for the examination of nine hub lncRNAs’ co-expressed genes based on the TCGA database, and the thresholds were set at the absolute value of correlation coefficient ≥ 0.5 as well as *p* < 0.05. To clarify the potential mechanisms of the nine-lncRNAs signature, we subsequently performed gene set enrichment analysis (GSEA, http://software.broadinstitute.org/gsea/index.jsp) as the following thresholds: false discovery rate (FDR) < 0.25 & *p* < 0.05.

### Analysis of Gene Expression and Tumor-Infiltrating Immune Cells

CIBERSORT algorithm was utilized to calculate tumor infiltrating immune cells, according to on gene expression predicting the proportions of different immune cells types in the gene expression profiles. The correlation between immune cells and risk score was evaluated by R software “vioplot” package.

TIMER (https://cistrome.shinyapps.io/timer/) was applied to explore the correlation between the hub lncRNAs and tumor infiltrating immune cells (B cell, CD4 T cell, CD8 T cell, Dendritic cell, Macrophage, Neutrophil), relying on 10,897 clinical specimens retrieved from the TCGA database [29].

### Construction of Diagnostic Model of hub lncRNAs

The logistic regression analysis was utilized to screen important hub lncRNAs and establish a diagnostic model based on the expression values in normal and PTC samples. ROC and calibration curve (1,000 bootstrap resamples) were utilized to assess the performance of the diagnostic model. PCA was conducted to detect the effectiveness of the diagnostic model with six important lncRNAs.

### Statistics

SPSS version 22.0 (IBM Corporation, Armonk, NY, United States) and GraphPad Prism 8.0 (SanDiego, CA, United States) were utilized to analyze the statistical data. Kaplan-Meier method was employed to construct the overall survival curves. The univariate and multivariate Cox regression analyses were implemented to determine the independent prognostic indicators. The relevance of signature and clinical parameters were assessed by Chi-square test. *p* < 0.05 was considered as a cutoff value in all circumstances.

## Results

### Clinical Characteristics of Patients

**FIGURE 1:**
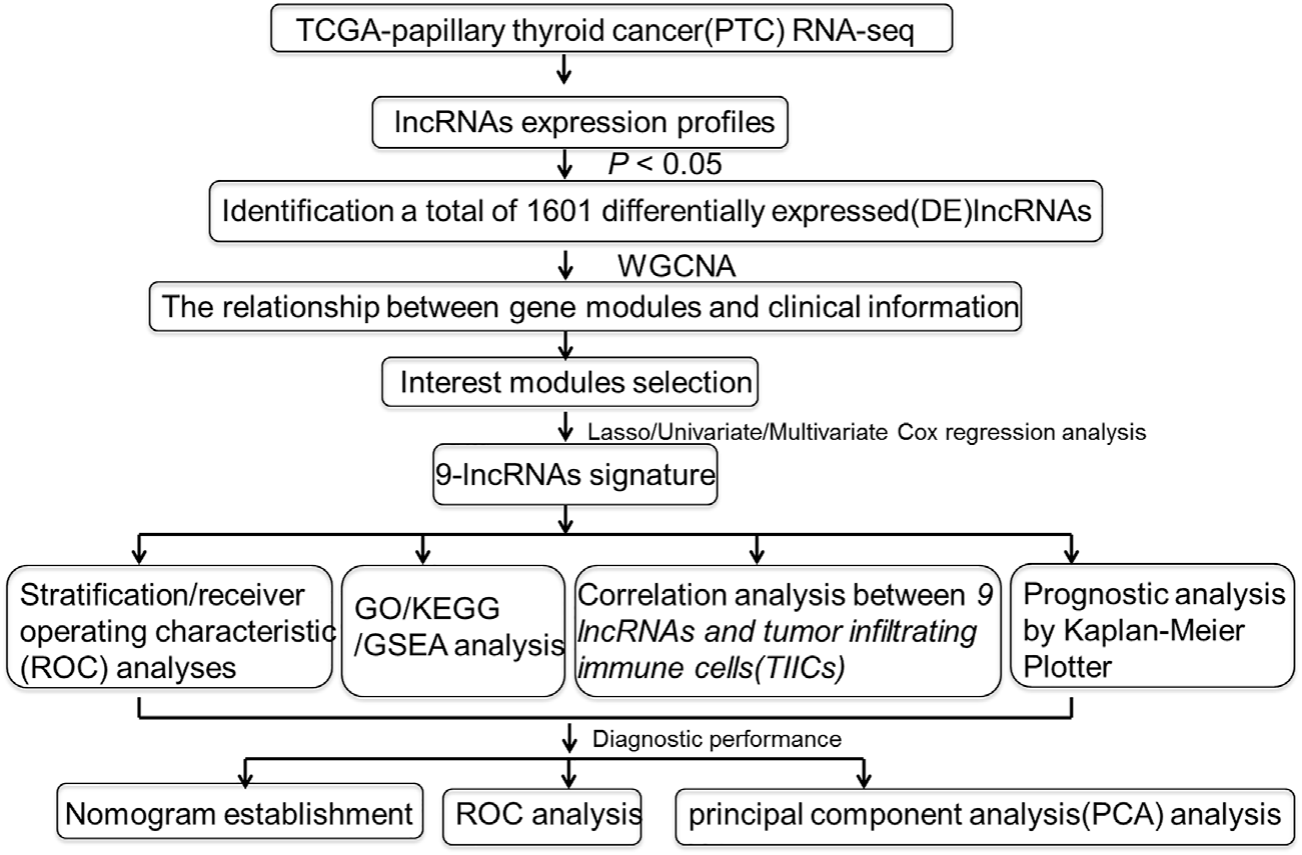
Flowchart of the study.

The schematic diagram of our present study was exhibited in Figure 1. Clinical data derived from TCGA database that contains a total of 448 primary PTC patients whose follow-up time over 30 days was collected for the present study. The detailed information of 448 PTC patients was summarized in Table 1. In Table 1, slightly more than half of the patients (56.25%) was aged over 45 years, and the vast majority of PTC patients were female which accounted for 73.88%. The vital status of most patients (96.65%) was alive. Furthermore, 66.96% of PTC patients were at an early stage of cancer as well as T/N/M stage (T1/T2, 62.72%; M0, 98.44%). Additionally, a total of 56 healthy volunteers were enrolled as the normal group, whose age and gender were matched (Supplementary Table S1).

### Co-Expression Network Construction and key Module Determination

**FIGURE 2:**
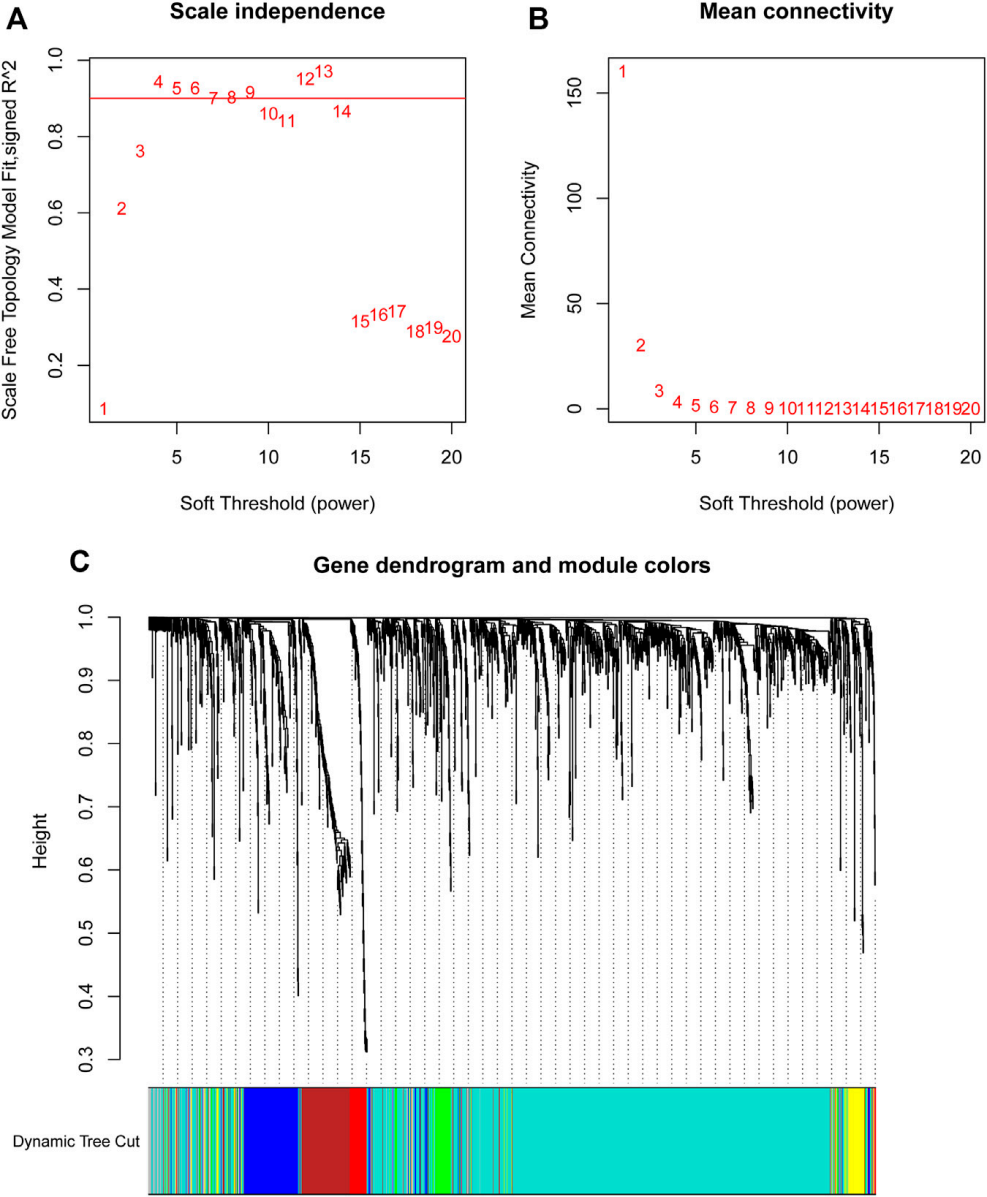
Soft-thresholding values estimation and clustering dendrograms. Analyses of the scale-free fit index **(A)** and the mean connectivity **(B)** for various soft-thresholding powers. **(C)** Dendrogram of all differentially expressed lncRNAs clustered on the basis of the measurement of dissimilarity.

**FIGURE 3:**
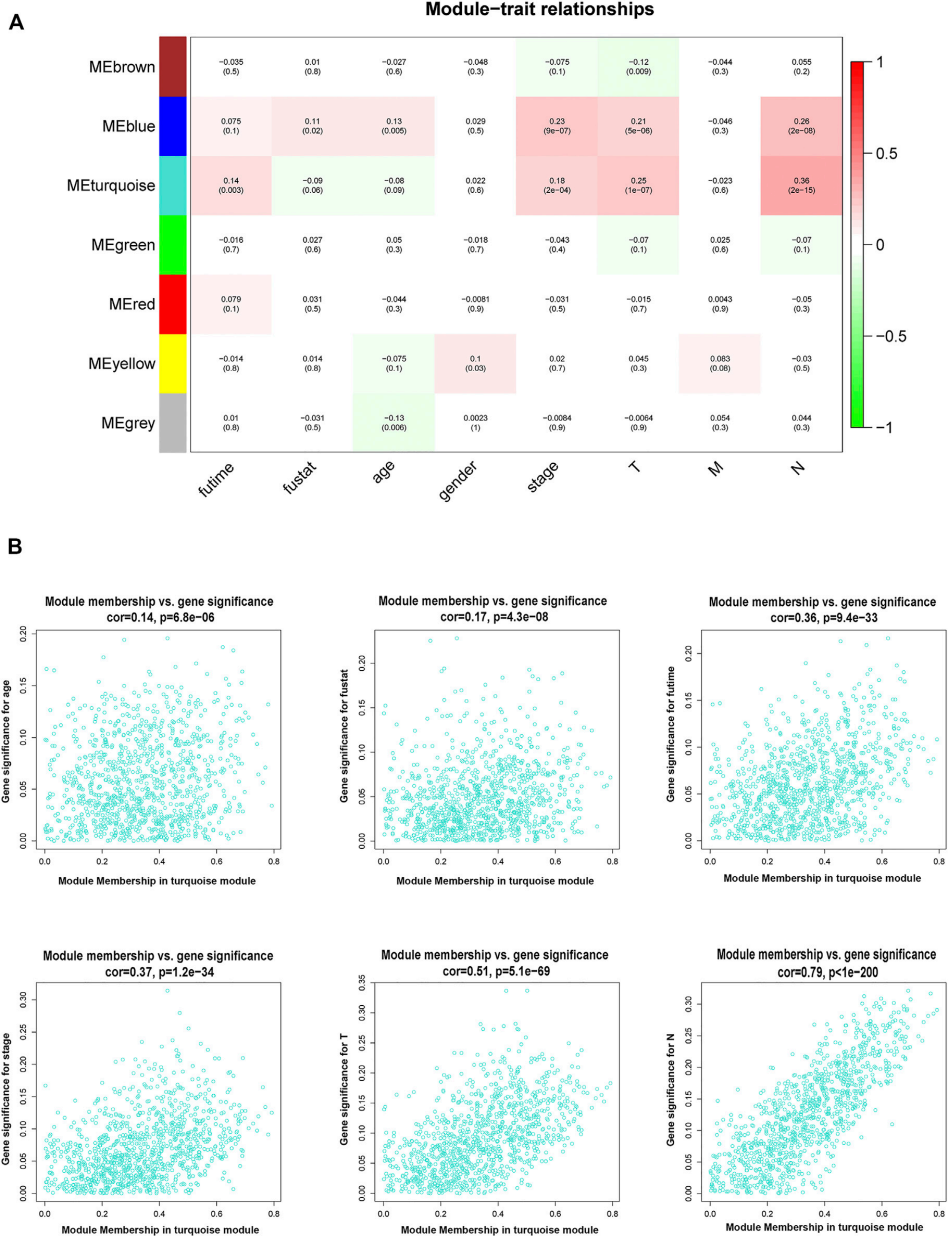
Association between modules and clinical traits. **(A)** Heatmap of the relationship between the module eigengenes and clinical traits of PTC. We selected the turquoise module for the following analysis. **(B)** The scatter plot between the turquoise module membership and the gene significance for age, survival status, survival time, stage, T, M, and N. T denotes primary tumor, M denotes distant metastasis, and N denotes lymph node metastasis. PTC, papillary thyroid cancer.

By using the statistical software R and “edgeR” package, the remarkable DElncRNAs were screened by comparing the PTC tumor cases with normal samples. In total, 1,601 DElncRNAs were identified, including 1,000 upregulated DElncRNAs and 601 down-regulated DElncRNAs. To determine the key modules that most related with clinical traits of PTC, WGCNA on the 1,601 DElncRNAs and 448 samples with complete information was performed. By setting soft-thresholding power and cutting height as 0.25, a total of seven key modules were achieved (Figures 2A–C). In the heatmap of module-trait correlations, we observed that the blue and turquoise modules were highly related with clinical traits (Figure 3A). As the turquoise module possessed the largest number of DElncRNAs and a higher correlation with certain clinical traits (such as T, N, and survival time) relative to the blue module, we selected turquoise module for further analysis. The exact associations between genes of turquoise module and clinical features of PTC patients were revealed in Figure 3B.

### Validation of hub lncRNAs

**FIGURE 4:**
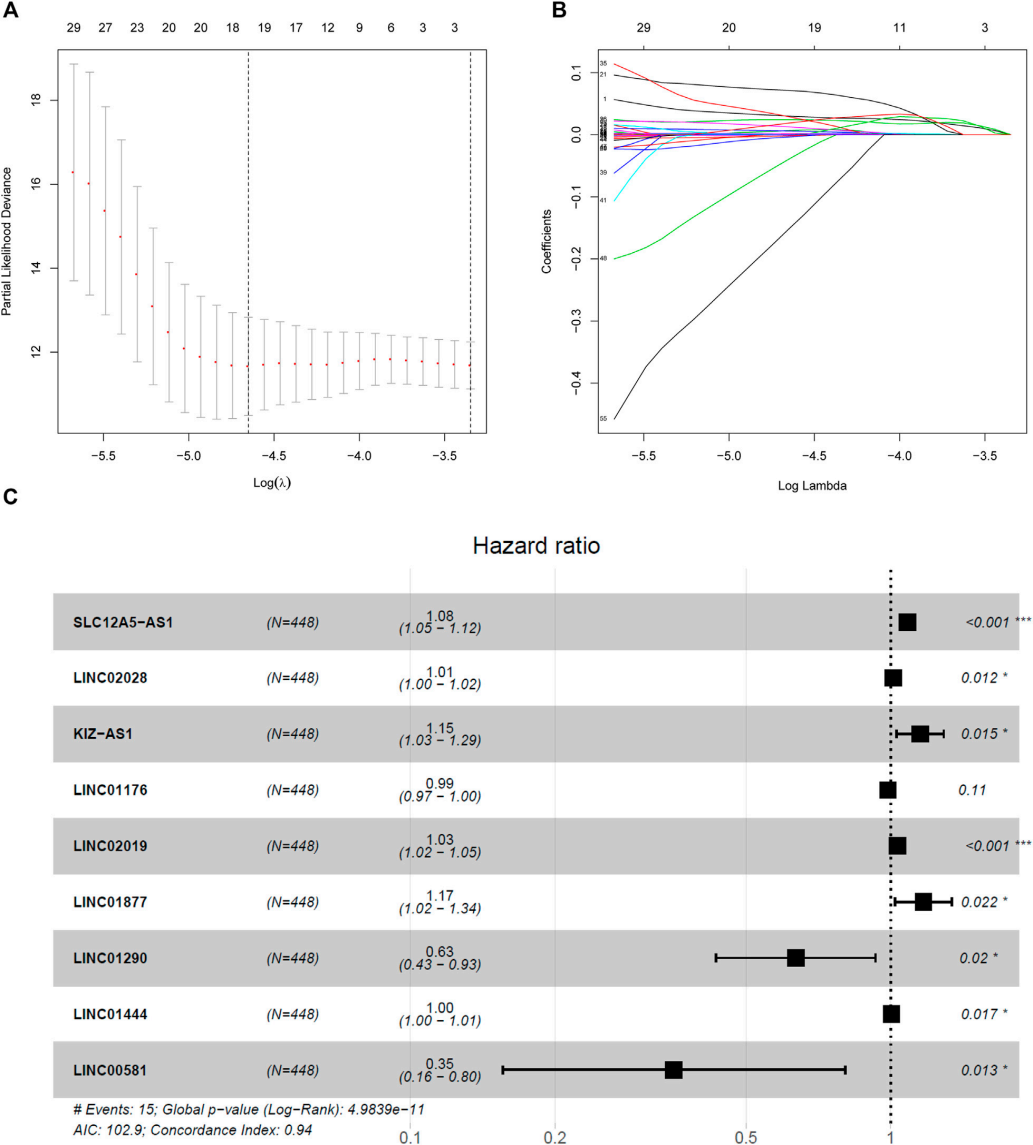
Nine-lncRNAs were identified by LASSO Cox regression. **(A)** LASSO coefficient profiles of the fractions genes in the turquoise module. **(B)** Tenfold cross-validation for tuning parameter selection in the LASSO model. **(C)** Forest plot of nine hub lncRNAs.

A LASSO Cox regression model was constructed and shown in Figures 4A,B and 9 hub lncRNAs were eventually identified, including six high-risk lncRNAs SLC12A5-AS1, LINC02028, KIZ-AS1, LINC02019, LINC01877, LINC01444 and three low-risk lncRNAs LINC01176, LINC01290 and LINC00581 (Figure 4C). The risk score was calculated for the nine-lncRNAs signature as follows:

Risk score=(0.081*the expression level of “SLC12A5-AS1”)+(0.014*the expression level of LINC02028)+(0.141*the expression level of “KIZ-AS1”)+(−0.013*the expression level of “LINC01176”)+(0.033*the expression level of “LINC02019”)+(0.158*the expression level of “LINC01877”)+(−0.455*the expression level of “LINC01290”)+(0.005*the expression level of “LINC01444”) (−1.039*the expression level of “LINC00581”).

**FIGURE 5:**
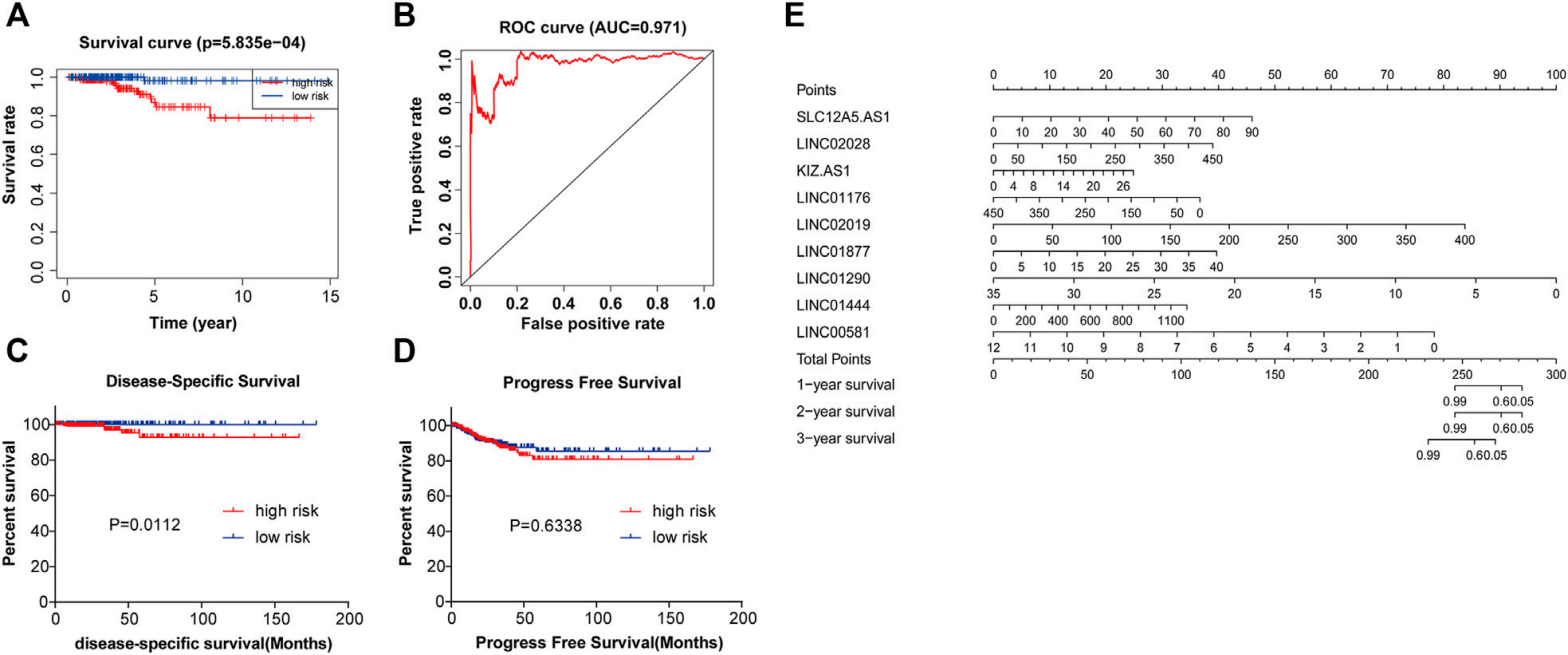
The nine-lncRNAs signature has significant predictive value in PTC. **(A)** The Kaplan-Meier method was used to plot the overall survival curve for the high risk and low risk groups, *p* = 5.835e-04. **(B)** ROC curve of nine-lncRNAs signature in PTC, AUC = 0.971. **(C)** DSS of PTC patients in high- and low-risk groups, *p* = 0.0112. **(D)** PFS of PTC patients in high- and low-risk groups, *p* = 0.6338. **(E)** Nomogram for predicting the 1-, 2-, and 3-years survival of PTC patients with nine-lncRNAs signature. AUC, area under the curve; DSS, disease-specific survival time; PFS, progression-free survival time.

Based on the median value of risk score, 448 PTC patients were classified into two groups: high risk group (*n* = 224) and low risk group (*n* = 224). Compared with the low risk group, PTC patients of high risk group showed the poorer outcomes (Figure 5A). Death was taken as the end point for overall survival analysis. The ROC analysis was performed to examine the prognostic value of nine-lncRNAs signature. The area under curve (AUC) of nine-lncRNAs signature was 0.971 (Figure 5B). Results showed that patients in the high risk group had lower Disease-specific survival (DSS) than those in the low risk group (*p* = 0.0112) (Figure 5C). DSS refers specifically to death due to thyroid cancer as the study end point. Progression-free survival (PFS) of PTC patients was not statistically different between high risk and low risk groups. However, as can be seen from Figure 5D, the PFS of the high risk group was also lower than that of the low risk group. PFS took the appearance of disease progression as the end point. The nomogram indicated that the nine-lncRNAs signature may serve as a prognostic candidate for PTC (Figure 5E).

### Clinical Correlation of Signature in PTC

To better illustrate the clinical significance of this model, we analyzed the correlation between risk score and clinical features, including age, gender, pathological stage, T/N/M stage, and survival status. The results exhibited that age, pathological stage, T stage, and survival status of PTC patients were correlated with the risk score with statistical significance (Table 2). Furthermore, the number of patients with high risk scores in advanced pathological stage and T stage was more than that of patients with low risk scores. These findings confirmed that this signature was highly related with clinical features of PTC patients.

**TABLE 2:** Correlation of risk score of signature and clinical characteristics of PTC patients.

Characteristics	Risk		*p* value
	Low	High	
**Age**			<0.0001
<45	122	74	
≥45	102	150	
**Gender**			0.067
female	174	157	
male	50	67	
**Pathologic Stage**			<0.0001
I	149	99	
II	24	28	
III	38	62	
IV	13	35	
**T stage**			<0.0001
T1	78	56	
T2	81	66	
T3	62	85	
T4	3	17	
**N stage**			0.343
N0	115	125	
N1	109	99	
**M stage**			0.057
M0	223	218	
M1	1	6	
**Death**			0.006
No	222	211	
Yes	2	13	

### The Nine-lncRNAs Signature is an Independent Prognostic Factor in PTC

**FIGURE 6:**
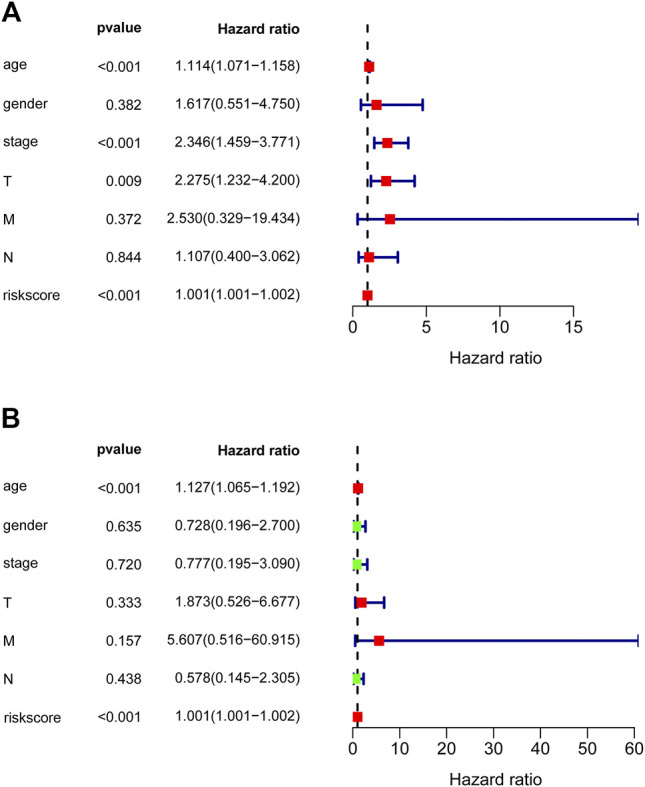
Forest plot of overall survival analyses. **(A)** Univariate and **(B)** multivariate analyses were utilized to assess whether the nine-lncRNAs signature is an independent factor in PTC. The red or green solid squares indicate the values of HR, and the transverse lines with dark blue indicate 95% CI. HR denotes hazard ratio. CI denotes confidence interval.

To detect whether the prognostic significance of nine-lncRNAs signature is depended on the clinical parameters, univariate and multivariate Cox regression analyses were adopted to analyze the following variables like nine-lncRNAs signature’s risk score, age, gender, pathological stage, and T/N/M stage. In univariate Cox analysis, risk score (HR = 1.001, *p* < 0.001), age (HR = 1.114, *p* < 0.001) and pathological stage (HR = 2.346, *p* < 0.001) were all powerful variables related with prognosis of PTC patients in TCGA-PTC cohort (Figure 6A). Furthermore, results of multivariate Cox analysis demonstrated that risk score (HR = 1.001, *p* < 0.001) and age (HR = 1.127, *p* < 0.001) were still proved to be independent factors based on the TCGA-PTC cohort (Figure 6B). All findings elucidated that the nine-lncRNAs signature might function as an independent prognostic factor in PTC.

### Function Annotation of the hub lncRNAs and Nine-lncRNAs Signature

**FIGURE 7:**
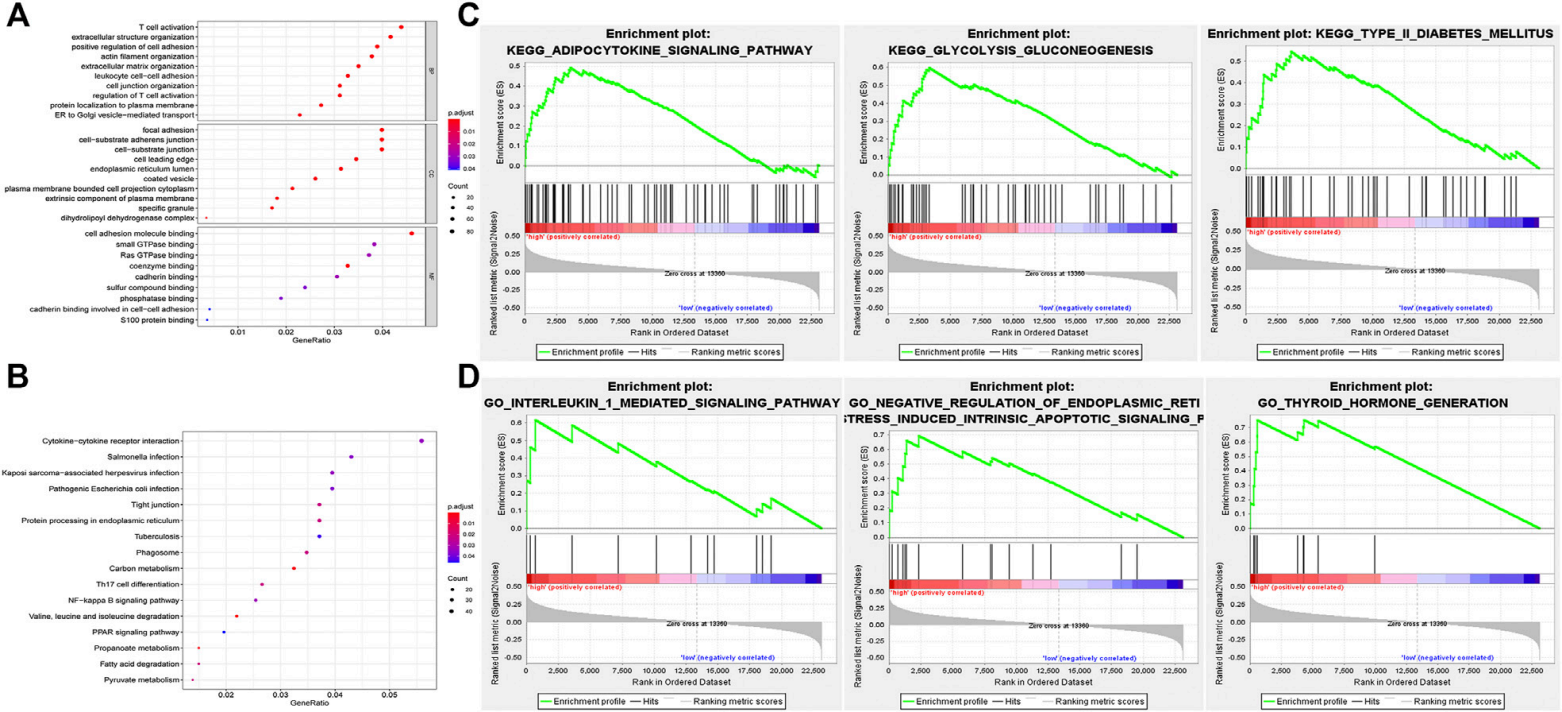
Functional enrichment analyses of nine key lncRNAs. **(A)** Top 10 GO BP terms were enriched using GO for the indicated nine lncRNAs. **(B)** Top 16 pathways were enriched by KEGG analysis. **(C)** Only three enriched KEGG pathways are listed that related to the high risk of the model. **(D)** Three GO terms were identified that significantly correlated with PTC. GO, gene ontology; BP, biological process; KEGG, Kyoto Encyclopedia of Genes and Genomes; GSEA, gene set enrichment analysis.

In order to predict the possible biological processes (BP) and signaling pathways that the nine lncRNAs may participate in, functional enrichment analyses, GO, and KEGG analyses were performed based on the co-expressed genes of nine hub lncRNAs in PTC. The top 10 GO BP terms with highest enrichment were exhibited in Figure7A, including T cell activation, positive regulation of cell adhesion, focal adhesion, cell adhesion molecule binding, etc. (*p* < 0.05). In addition, the top 16 pathways that were enriched by the nine lncRNAs in KEGG analysis were presented in Figure7B, including Cytokine-cytokine receptor interaction, NF-kappa B signaling pathway, and Th17 cell differentiation (*p* < 0.05).

In addition to GO and KEGG analyses, GSEA was also implemented to inquire into the potential biological processes and signaling pathways of the nine-lncRNAs signature (Figures 7C,D). Eighteen KEGG pathways were enriched. Among these, three pathways related to PTC pathogenesis, namely adipocytokine signaling pathway, glycolysis and gluconeogenesis, and type_II_diabetes mellitus, were enriched in the high risk group, indicating that the nine-lncRNAs signature may be implicated in the progression of PTC (Figure 7C, *p* < 0.05). Meanwhile, a total of 782 GO terms were identified and the pathways-related with PTC development were as follows: interleukin 1 mediated signaling pathway, negative regulation of endoplasmic reticulum stress induced intrinsic apoptotic signaling pathway, thyroid hormone generation (Figure 7D, *p* < 0.05).

### Comparisons of Tumor-Infiltrating Immune Cells in High/low Risk Group and Correlation of Nine hub lncRNAs Expression With Tumor-Infiltrating Immune Cells

**FIGURE 8:**
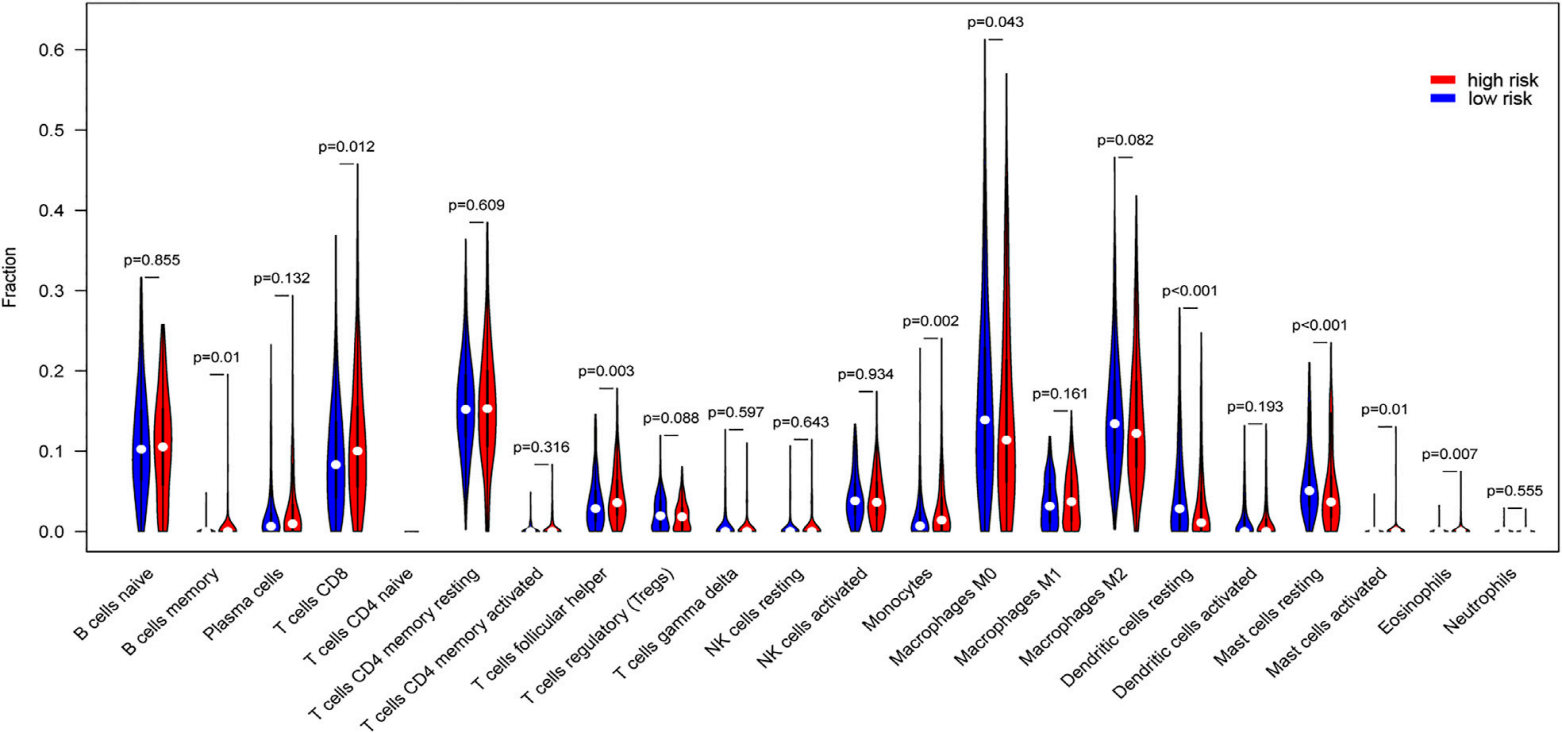
Differences of tumor-infiltrating immune cells in high/low risk score group. Proportions of 22 types of tumor-infiltrating immune cells in high risk score and low risk score groups were compared.

The tumor microenvironment is composed of tumor cells, stromal cells and infiltrating immune cells. Thus, we tested proportions of 22 types of immune cells in the high/low risk group. Memory B cells, CD8 T cells, follicular helper T cells, monocytes, activated mast cells, and eosinophils were higher in risk than the low-risk group, while M0 macrophages, resting dendritic cells, and resting mast cells were lower in risk than the low-risk group (Figure 8). These findings showed different relationships between signatures and different types of infiltrating immune cells, resulting in different survival outcomes.

Then, we used TIMER to determine the associations between expressions of nine hub lncRNAs and tumor infiltrating immune cells. As illustrated in Supplementary Figure S1, except for KIZ-AS1, all other lncRNAs were correlated with the content of more than two kinds of immune cells, which was statistically significant.

### Survival Analysis of Nine hub lncRNAs in PTC

**FIGURE 9:**
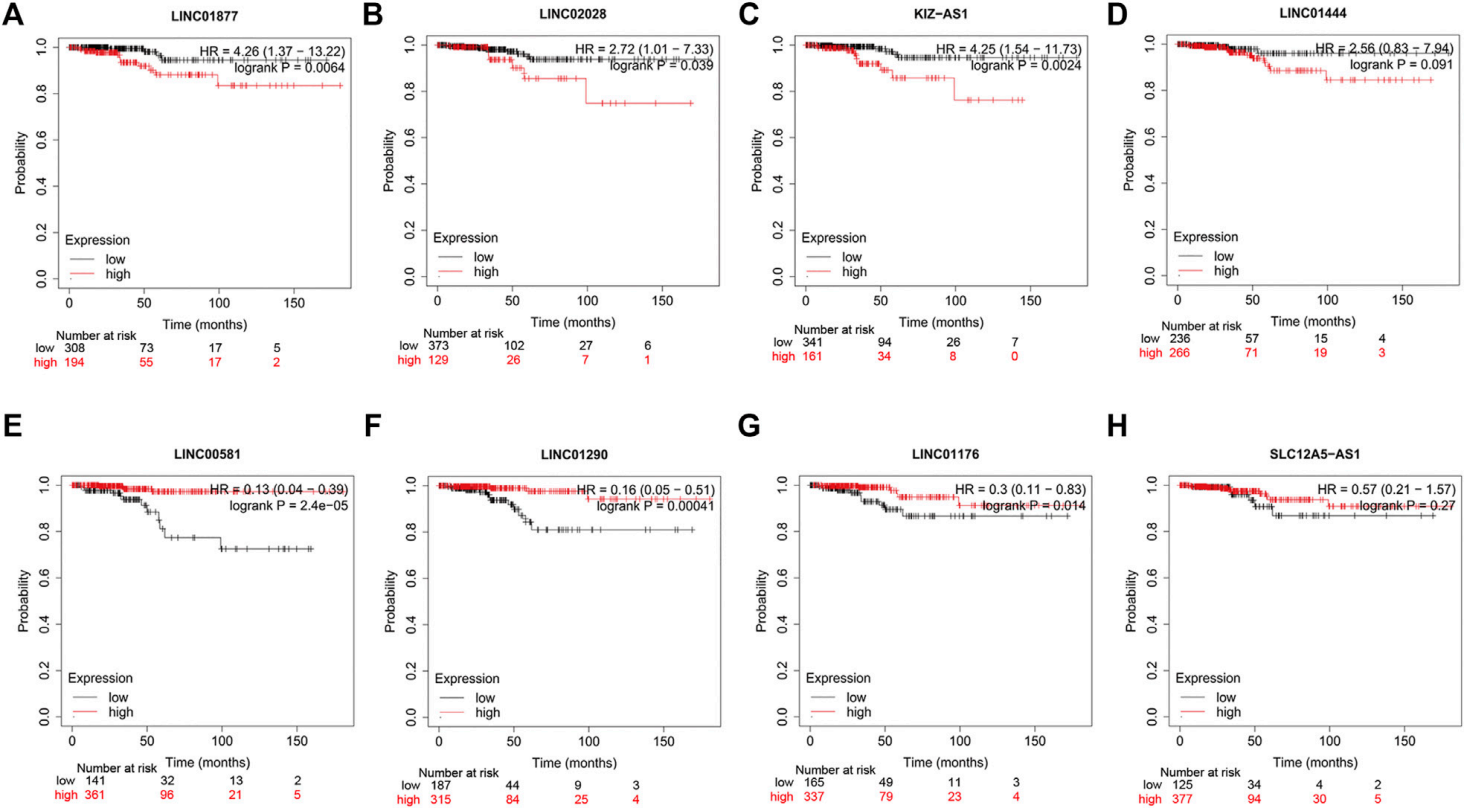
Survival analysis of eight hub lncRNAs using KM Plotter website. **(A)** LINC01877, **(B)** LINC02028, **(C)** KIZ-AS1, **(D)** LINC01444, **(E)** LINC00581, **(F)** LINC01290, **(G)** LINC01176, and **(H)** SLC12A5-AS1. Red lines represent high expression of the eight hub lncRNAs and black lines represent low expression.

As previously described, results of the multivariate Cox regression analyses disclosed that SLC12A5-AS1, LINC02028, KIZ-AS1, LINC02019, LINC01877, and LINC01444 were high-risk genes, while LINC01176, LINC01290, and LINC00581 were low-risk genes (Figure 4C; Supplementary Table S2). To further detect the prognostic significances of nine hub lncRNAs in PTC, we accessed the KM Plotter website. The high expressions of LINC01877, LINC02028, and KIZ-AS1 were linked with the unfavorable outcomes of PTC patients, and low expressions of LINC00581, LINC01290 and LINC01176 were related with poor outcomes, with statistical significance (Figure 9, *p* < 0.05). There was no data of LINC02019 in the KM Plotter website. In summary, these data provide some support for the predictable role of nine hub lncRNAs in PTC.

### Diagnostic Nomograms Establishment and Performance Evaluation

**FIGURE 10:**
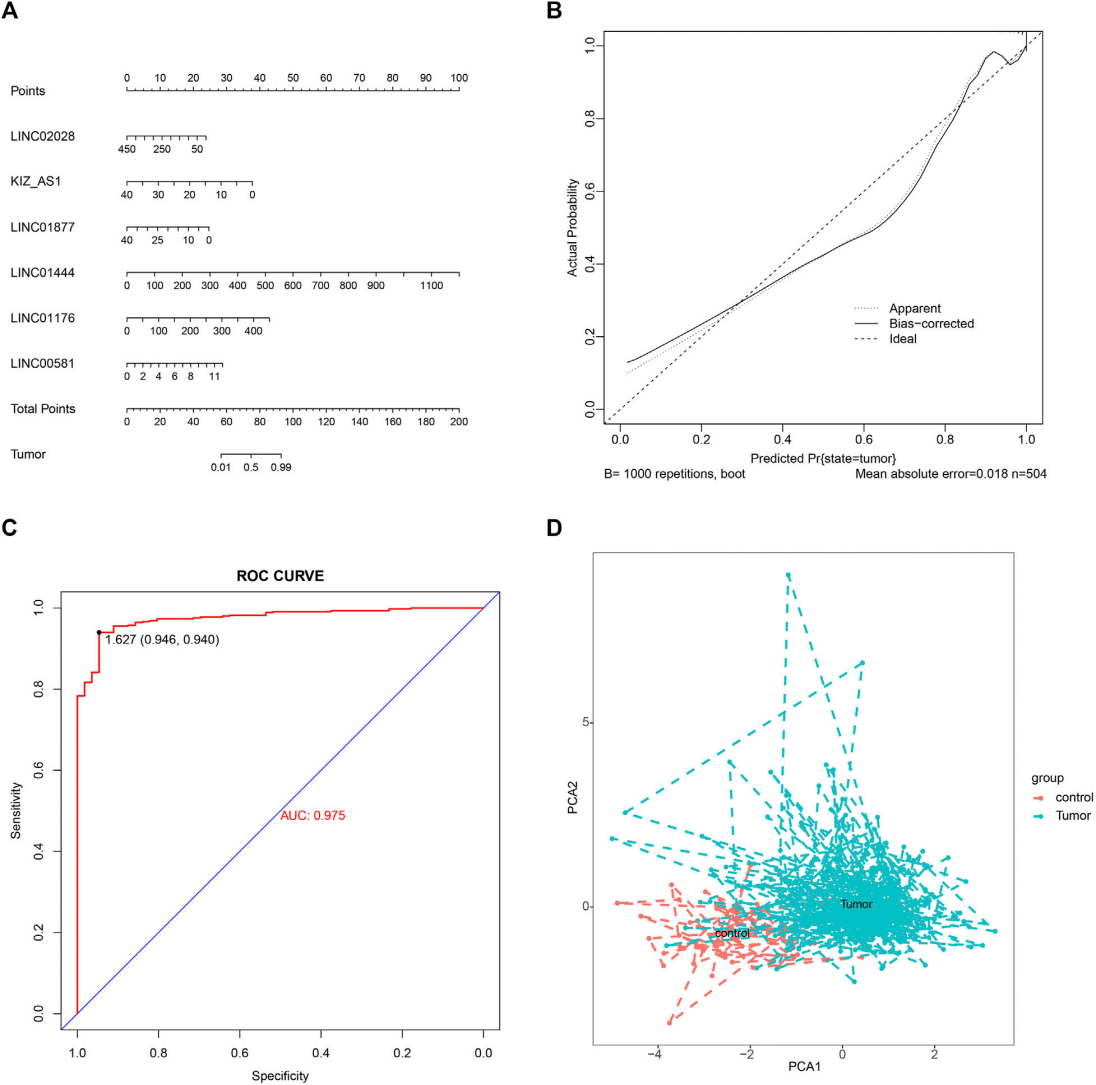
Establishment of nomograms and performance evaluation. **(A)** Nomogram for predicting the diagnostic significance of six important lncRNAs in PTC. **(B)** Calibration plot of the nomogram. **(C)** AUC was calculated from the ROC curve. **(D)** PCA cluster plot of six important lncRNAs expression in tumor versus control samples. ROC, receiver operating characteristic; PCA, principal component analysis.

To build a potential diagnostic model, we first performed the logistic regression analysis for the identification of important hub lncRNAs. Through screening, six important hub lncRNAs were identified, including LINC02028, KIZ-AS1, LINC01877, LINC01444, LINC01176, and LINC00581. On the basis of these six important lncRNAs, a nomogram was built (Figure 10A). The calibration curve showed high consistence between actual probability and predicted survival proportion (Figure 10B). The AUC of the diagnostic model was 0.975, which suggested that the nomogram might have high sensitivity for PTC diagnosis (Figure 10C). PCA further confirmed the effectiveness of the diagnostic model with six important lncRNAs, and the results showed that the normal and tumor samples can be better distinguished based on the expression level of six important lncRNAs (Figure 10D).

### Comparison of Prognostic Prediction Score Between our Signature and Published Signatures in PTC or TC

**FIGURE 11:**
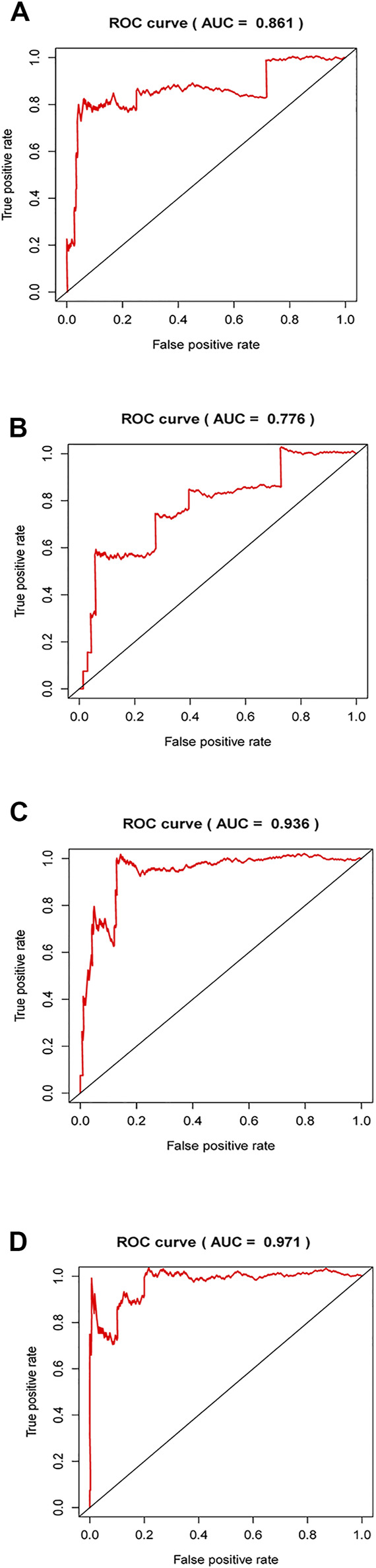
Comparison of ROC curve in published lncRNAs signatures and our model. **(A–C)**, ROC curves were plotted by using published lncRNAs signatures in other previous studies. **(D)**, ROC curves were presented in accordance with established risk lncRNA signature in our present work.

By retrieving the published lncRNA signature in others’ studies, we chose three signatures, including six-lncRNA signature [30], seven-lncRNA signature [31] and nine-lncRNA signature [32], and performed ROC evaluation to assess the prognostic prediction performance based on these lncRNAs. The results exhibited in Figure 11 showed that AUC values of these three signatures were 0.861, 0.776, and 0.936, all lower than AUC value of our model (0.971). The data indicates a favorable potential of our signature for predicting the prognosis of PTC.

## Discussion

PTC, as the most common malignancy in the endocrine system, is a great challenge for public health [33]. Thus, it is of great urgency to identify specific cancer-related biomarkers for risk evaluation, which could be employed to predict the prognosis of PTC patients and promote the development of effective therapies to PTC. In the investigation, a systematic bioinformatics analysis identified a nine-lncRNAs signature that significantly correlated with the pathogenesis of PTC.

WGCNA is a systematic biological algorithm that could distinguish highly synergistically altered genes and unearth relevance between genes and clinical characteristics [34]. Therefore, it has been widely utilized to identify the candidate biomarkers of diverse diseases, such as Alzheimer’s disease, NSCLC, colon cancer and PTC [35–38]. For example, a total of 11 genes have been reported to exert important roles in PTC relapse, which shed novel insights in the lymph node metastasis of PTC [39]. Han et al. determined that nine genes containing CDH5, KDR, CD34, FLT4, EMCN, FLT1, ROBO4, PTPRB, and CD93 could mediate the I^131^ radiotherapy in TC patients [40]. Three genes (NGF, FOS, and GRIA1) are found to be involved in regulation of the features of TC stem cells using WGCNA [41]. Additionally, for a long time in the past, genomic libraries of many non-protein transcripts, including lncRNAs, were considered as insignificant transcription “junk”. However, the implementation of TCGA program has focused a tremendous amount of attention on the roles of lncRNAs in tumorigenesis [42, 43]. Therefore, in the present study, to investigate possible prognostic lncRNAs for PTC, we downloaded the lncRNAs expression profiles from TCGA database and mined the underlying microarrays to perform a comprehensive analysis of lncRNA expression matrix files. In the TCGA-PTC cohort, a total of 1,601 DElncRNAs, 1,000 up-regulated lncRNAs and 601 down-regulated lncRNAs, were identified. Co-expression network construction and key modules identification through WGCNA identified seven modules, of which, the genes of turquoise module were all linked with age, survival status, survival time, stage, stage of tumor, and stage of metastasis.

After analyzing the genes of turquoise module by LASSO analysis, we finally obtained nine hub lncRNAs, including SLC12A5-AS1, LINC02028, KIZ-AS1, LINC02019, LINC01877, LINC01444, LINC01176, LINC01290, and LINC00581. The overall survival curve was plotted by Kaplan-Meier and revealed that PTC patients in the high risk group had unfavorable outcomes compared with the patients in the low risk group. The ROC analysis elucidated that the AUC of the identified nine-lncRNAs signature for overall survival rates was 0.971, indicating a satisfactory predictive value for PTC prognosis. The nomogram for 1-, 2- and 3-years overall survival suggested that the nine-lncRNAs signature was suitable for predicting the probability of overall survival of PTC patients. Furthermore, univariate and multivariate Cox regression analyses were adopted to identify the associations between following factors like risk score, age, gender, stage and T/N/M stage and PTC prognosis, which demonstrated that the nine-lncRNAs signature may be an independent candidate for predicting the prognosis of PTC patients.

Notably, up to date, there are fe reports regarding the biological roles of nine hub lncRNAs. Therefore, the results of the functional enrichment analyses could further promote the understanding of potential functions of nine hub lncRNAs. In GO and KEGG analyses, 2036 mRNAs that co-expressed with the nine hub lncRNAs were enriched in 228 GO terms and 16 pathways, including T cell activation, positive regulation of cell adhesion, focal adhesion, cell adhesion molecule binding, Cytokine-cytokine receptor interaction, NF-kappa B signaling pathway, and Th17 cell differentiation. As previously described, the activation of T cell intracellular antigen can suppress the development of anaplastic TC [44]. Song et al. indicated that the variants of genes-related with cell adhesion could be utilized to predict PTC aggressiveness [45]. NF-kappa B signaling pathway has been identified as a putative target for the treatment of advanced TC [46]. Besides, the underlying mechanism of nine-lncRNAs signature was detected using GSEA and 18 pathways were obtained, such as adipocytokine signaling pathway, glycolysis and gluconeogenesis, type II diabetes mellitus as well as 782 GO terms (for instance, interleukin 1 mediated signaling pathway, negative regulation of endoplasmic reticulum stress induced intrinsic apoptotic signaling pathway, thyroid hormone generation). It is well known that adipokines could regulate body mass and affect glucose homeostasis, cell proliferation, and other important cell procedures, which are all involved in tumor growth. Moreover, the deregulation of adipokines is observed in the head and neck cancer [47]. The energy provision of tumor cells relies nearly entirely on glycolysis and gluconeogenesis [48]. A previous report suggests that early type II diabetes mellitus might have a protective effect against TC [49]. Interleukin 1 is involved in the progression of medullary TC via mediating other essential pathways [50]. These findings of functional enrichment analyses determined that the nine hub lncRNAs may be required for PTC by modulating various signaling pathways and biological processes.

Tumor microenvironment contains tumor cells and tumor infiltrating immune cells. In order to explore the relationship of this signature and immune infiltration, we assessed the differences of 22 types of tumor infiltrating immune cells in the high/low risk score group. The results displayed that memory B cells, CD8 T cells, follicular helper T cells, monocytes, activated mast cells, eosinophils were higher in high-risk than low-risk group, while M0 macrophages, resting dendritic cells, and resting mast cells were lower in risk than the low-risk group. In the tumor environment, mast cells stimulate angiogenesis resulting in the growth and development of tumors [51]. Monocytes can indirectly promote extracellular matrix degradation and tumor angiogenesis, thereby promoting tumor progression and metastasis [52]. Studies have found that low CD4+/CD8 + ratio is related to a poor survival of tumor patients [53]. To further investigate the possible functions of this signature, nine hub lncRNAs, we referred to the TIMER website and found that except for KIZ-AS1, all other lncRNAs were correlated with the content of more than two kinds of immune cells with significant differences. These suggested that the functions of this signature composed of nine hub lncRNAs related to immunological regulation of the tumor microenvironment.

Additionally, the diagnostic model was then built using logistic regression, containing LINC02028, KIZ-AS1, LINC01877, LINC01444, LINC01176, and LINC00581. Calibration plot and ROC curves unearthed the high accuracy and sensitivity of this diagnostic model, which was enhanced by PCA analysis.

Nevertheless, a few limitations of this present study needed to be noted. Firstly, because all of the clinical samples were derived from TCGA database, we might overlook other potential lncRNAs candidates. A larger sample size should be used to validate the results of this study. Secondly, the data of LINC02019 in KM Plotter website were missing, therefore PTC samples should be harvested in the future for further validation of nine hub genes’ potential role in PTC. Thirdly, the *in vitro* and vivo functional experiments are required to verify their accurate biological effects. Fourth, the tumor microenvironment of PTC patients has been proven to be affected by concomitant Hashimoto Thyroiditis(HT). The coexistence of HT is related with the inf immunophenotype, whereas PTC patients without HT are mainly characterized by immune desert (ID) or immune-excluded (IE) immunophenotype [54]. Thus, further research should be performed to explore the relationship between PTC and HT as well as the accurate phenotype of immune cells.

## Conclusion

In conclusion, a nine-lncRNAs signature was constructed in our present study, which might be an independent prognostic variable for patients with PTC and possess potential value for prognosis and risk assessment. A predictive model with high accuracy and sensitivity was also built for PTC diagnosis. These findings would advance our understanding of PTC pathogenesis and optimize the strategies for improving prognosis of PTC.

## Data Availability

The original contributions presented in the study are included in the article/Supplementary Material, further inquiries can be directed to the corresponding author.

## Supplementary Material

Supplementary Figure 1 Relevance of eight lncRNAs’ expression with tumor-infiltrating immune cells in PTC, including **(A)** SLC12A5-AS1, **(B)** LINC01176, **(C)** LINC02028, **(D)** LINC02019, and **(E)** LINC01290, **(F)** LINC01877, **(G)** LINC01444, and **(H)** LINC00581.
