# Supplementary material for: A Potential Nine-lncRNAs Signature Identification and Nomogram Diagnostic Model Establishment for Papillary Thyroid Cancer
Source: Pathol Oncol Res. 2022 Feb 23;28:1610012. doi: 10.3389/pore.2022.1610012 (PMC8906208; doi:10.3389/pore.2022.1610012)
Supplement: Supplementary file 1 [file DataSheet1.doc]

**Table S1.** **There was no significant difference in the age and gender between papillary thyroid cancer patients and normal control group.**

| **Characteristics** | **Tumor（n=448）** | **Control（n=56）** | **P-value** |
| --- | --- | --- | --- |
| **Age** |  |  | 0.164 |
| <45 | 196 | 30 |  |
| ≥45 | 252 | 26 |  |
| **Gender** |  |  | 0.694 |
| Female | 331 | 40 |  |
| Male | 117 | 16 |  |

Note: P>0.05.

**Table S2. Expressional pattern of nine hub lncRNAs in high- and low-risk groups in PTC.**

| **lncRNA** | **logFC** | **logCPM** | **PValue** | **FDR** |
| --- | --- | --- | --- | --- |
| SLC12A5-AS1 | 0.523952138 | 4.123072758 | 1.39E-05 | 0.000105279 |
| LINC02028 | 0.786825146 | 6.315853298 | 1.47E-14 | 1.27E-12 |
| KIZ-AS1 | 0.385772279 | 3.43885376 | 3.29E-05 | 0.000227572 |
| LINC01176 | -0.544377772 | 7.330511622 | 3.23E-11 | 1.18E-09 |
| LINC02019 | -0.123275975 | 6.588443519 | 0.085920178 | 0.166423403 |
| LINC01877 | 0.626885507 | 2.624474549 | 5.90E-05 | 0.000374109 |
| LINC01290 | -1.275272865 | 3.152923076 | 4.38E-35 | 1.60E-31 |
| LINC01444 | 0.686344453 | 6.943167245 | 6.43E-08 | 9.49E-07 |
| LINC00581 | -1.48827994 | 2.340471362 | 1.88E-31 | 2.75E-28 |

PTC, papillary thyroid cancer.
