# Supplementary figures and images for: A Potential Nine-lncRNAs Signature Identification and Nomogram Diagnostic Model Establishment for Papillary Thyroid Cancer
Source: Pathol Oncol Res. 2022 Feb 23;28:1610012. doi: 10.3389/pore.2022.1610012 (PMC8906208; doi:10.3389/pore.2022.1610012)

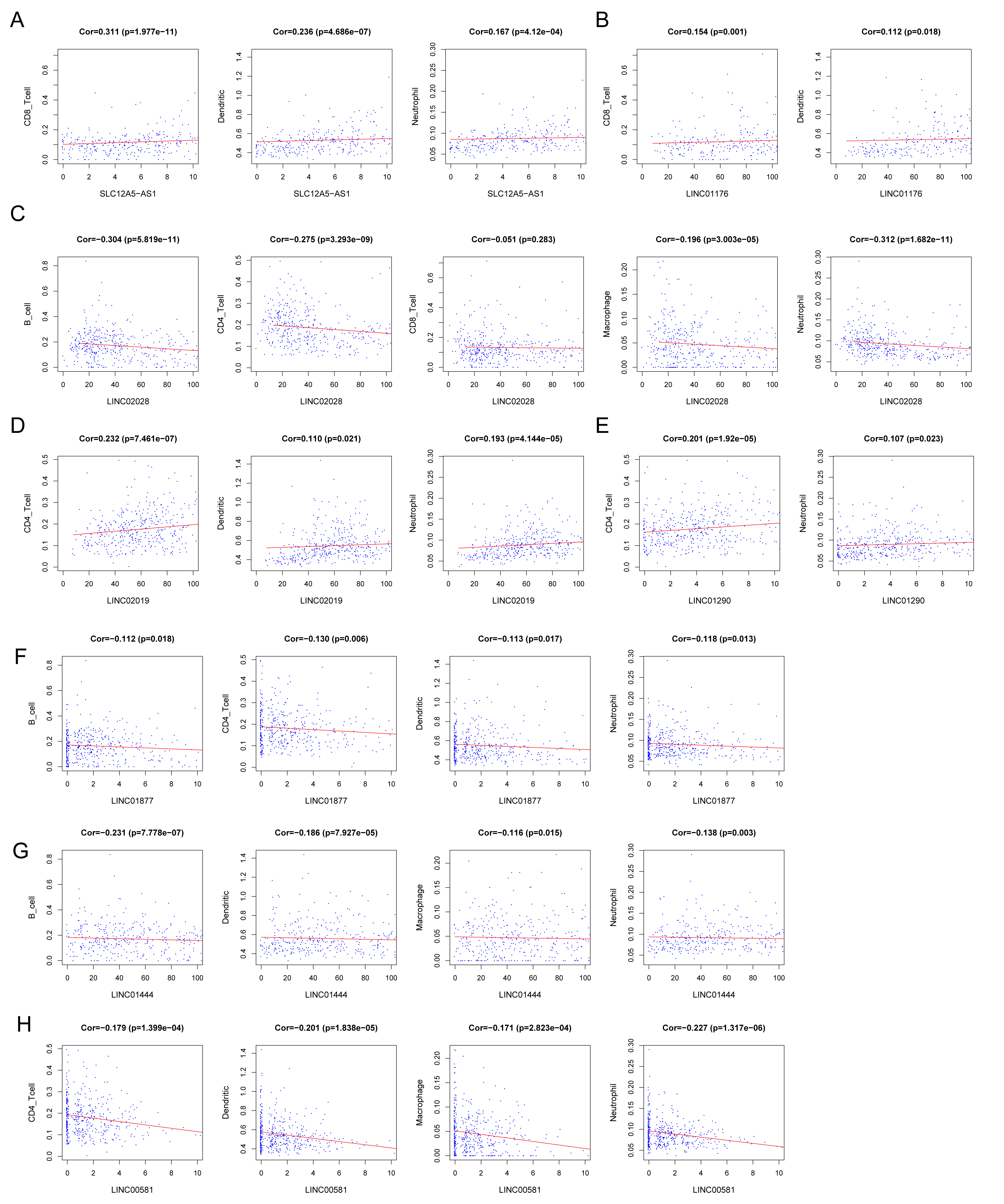

Supplement: Supplementary file 3 [file Image1.TIF]
